# Diagnostic accuracy of CT pulmonary angiography in suspected pulmonary hypertension

**DOI:** 10.1007/s00330-020-06846-1

**Published:** 2020-04-27

**Authors:** Andrew J. Swift, Krit Dwivedi, Chris Johns, Pankaj Garg, Matthew Chin, Ben J Currie, Alex MK Rothman, Dave Capener, Yousef Shahin, Charlie A Elliot, Thanos Charalampopolous, Ian Sabroe, Smitha Rajaram, Catherine Hill, Jim M. Wild, Robin Condliffe, David G. Kiely

**Affiliations:** 1 Department of Infection, Immunity and Cardiovascular Disease, University of Sheffield, Sheffield, UK; 2 INSIGNEO, Institute for In Silico Medicine, University of Sheffield, Sheffield, UK; 3 Academic Unit of Radiology, University of Sheffield, C Floor, Royal Hallamshire Hospital, Glossop Road, Sheffield, S10 2JF UK; 4 Sheffield Pulmonary Vascular Disease Unit, Royal Hallamshire Hospital, Sheffield Teaching Hospitals NHS Foundation Trust, Sheffield, UK; 5 Radiology Department, Sheffield Teaching Hospitals NHS Foundation Trust, Sheffield, UK

**Keywords:** Hypertension, pulmonary, Pulmonary artery, Heart ventricles, Diagnostic imaging

## Abstract

**Objectives:**

Computed tomography (CT) pulmonary angiography is widely used in patients with suspected pulmonary hypertension (PH). However, the diagnostic and prognostic significance remains unclear. The aim of this study was to (a) build a diagnostic CT model and (b) test its prognostic significance.

**Methods:**

Consecutive patients with suspected PH undergoing routine CT pulmonary angiography and right heart catheterisation (RHC) were identified. Axial and reconstructed images were used to derive CT metrics. Multivariate regression analysis was performed in the derivation cohort to identify a diagnostic CT model to predict mPAP ≥ 25 mmHg (the existing ESC guideline definition of PH) and > 20 mmHg (the new threshold proposed at the 6th World Symposium on PH). In the validation cohort, sensitivity, specificity and compromise CT thresholds were identified with receiver operating characteristic (ROC) analysis. The prognostic value of the CT model was assessed using Kaplan-Meier analysis.

**Results:**

Between 2012 and 2016, 491 patients were identified. In the derivation cohort (*n* = 247), a CT model was identified including pulmonary artery diameter, right ventricular outflow tract thickness, septal angle and left ventricular area. In the validation cohort (*n* = 244), the model was diagnostic, with an area under the ROC curve of 0.94/0.91 for mPAP ≥ 25/> 20 mmHg respectively. In the validation cohort, 93 patients died; mean follow-up was 42 months. The diagnostic thresholds for the CT model were prognostic, log rank, all *p* < 0.01.

**Discussion:**

In suspected PH, a diagnostic CT model had diagnostic and prognostic utility.

**Key Points:**

• *Diagnostic CT models have high diagnostic accuracy in a tertiary referral population of with suspected PH.*

• *Diagnostic CT models stratify patients by mortality in suspected PH.*

**Electronic supplementary material:**

The online version of this article (10.1007/s00330-020-06846-1) contains supplementary material, which is available to authorized users.

## Introduction

Pulmonary hypertension (PH) has been defined arbitrarily as a mean pulmonary artery pressure of at least 25 mmHg at rest [1]. However, data from normal subjects have suggested that a mean pulmonary artery pressure in excess of 20 mmHg is abnormal [2]. The 6th World Symposium on PH has therefore proposed a threshold of > 20 mmHg to define PH and a requirement for a pulmonary vascular resistance of at least 3 Wood Units to define pre-capillary PH. PH has many causes and its presence is associated with a high morbidity and a high mortality [3]. Due to the non-specificity of symptoms, PH is often diagnosed late. Given the availability of therapies for specific forms of PH, there is increasing interest in better patient phenotyping and improving diagnostic rates with imaging [4].

Transthoracic Doppler echocardiography is a non-invasive test, which is widely available, is relatively inexpensive and is recommended in international guidelines if the diagnosis of PH is suspected [1]. In a meta-analysis of diagnostic studies, the pooled sensitivity for the diagnosis of PH was 88% (84–92) and specificity was 56% (46–66) [5]. In patients with obesity or lung disease, views of the tricuspid regurgitant jet and cardiac chambers may be inadequate [6, 7]. A recent study has shown that, in a large population of patients undergoing echocardiography, tricuspid regurgitant jet velocity can be measured in 50% of patients [8]. More recently, there has been interest in the role of MRI techniques to identify patients with PH and a number of features visible on MRI such as elevated ventricular mass index [9–11], reduced pulmonary artery pulsatility [12, 13] and pulmonary flow [14, 15] may suggest the diagnosis of PH. However, MRI is expensive and less available than other imaging modalities. Right heart catheterisation (RHC) is the gold standard test for pressure measurement and thus diagnosis of PH.

Computed tomography has been seen to have several technological advances over the last three decades, and the introduction of iterative reconstruction CT algorithms has led to a significant reduction in image noise and radiation dose [16]. CT is frequently used in the evaluation of breathlessness and in the assessment of lung and increasingly cardiac disease [17–24]. The majority of studies using CT as a diagnostic tool have concentrated primarily on pulmonary artery size. However, remodelling of the cardiac chambers and bowing of the interventricular septum can also be detected on CT pulmonary angiography [18, 19, 25–27] in patients with PH. The aim of this study was to (a) build a diagnostic CT model in patients with suspected PH using the current guideline definition of PH (mPAP ≥ 25 mmHg) and the recent proposed definition of > 20 mmHg, and (b) test its prognostic significance.

## Materials and methods

### Patients

Consecutive treatment-naïve patients with suspected PH referred to a nationally designated PH centre (Sheffield Pulmonary Vascular Disease Unit) between April 2012 and March 2016 were identified from the ASPIRE Registry MRI database [28]. Inclusion criteria required CT pulmonary angiography to be performed within 90 days of RHC. All patients underwent MRI within 48 h of RHC. Ethical approval for this analysis of imaging techniques and routinely collected data was granted by our institutional review board.

### CT pulmonary angiography acquisition

All CT pulmonary angiograms in Sheffield were performed on a light-speed 64-slice MDCT scanner (GE Healthcare). Standard acquisition parameters were used: 100 mA with automated dose reduction, 120 kV, pitch 1, rotation time 0.5 s and 0.625 mm collimation. A 400 mm × 400 mm field of view was used with an acquisition matrix of 512 × 512. One hundred millilitres of intravenous contrast agent (Ultravist, Bayer) was administered at a rate of 5 mL/s. HRCTs were reconstructed using the contrast-enhanced acquisitions with 1.25 mm collimation from the apex of the lung to the diaphragm. Inclusion criteria for studies performed outside of Sheffield included CT pulmonary angiography with volumetric coverage of the pulmonary vasculature and cardiac structures and reconstructed slice thickness of 2 mm or less.

### CT image analysis

#### Vessel measurements

**Fig. 1:**
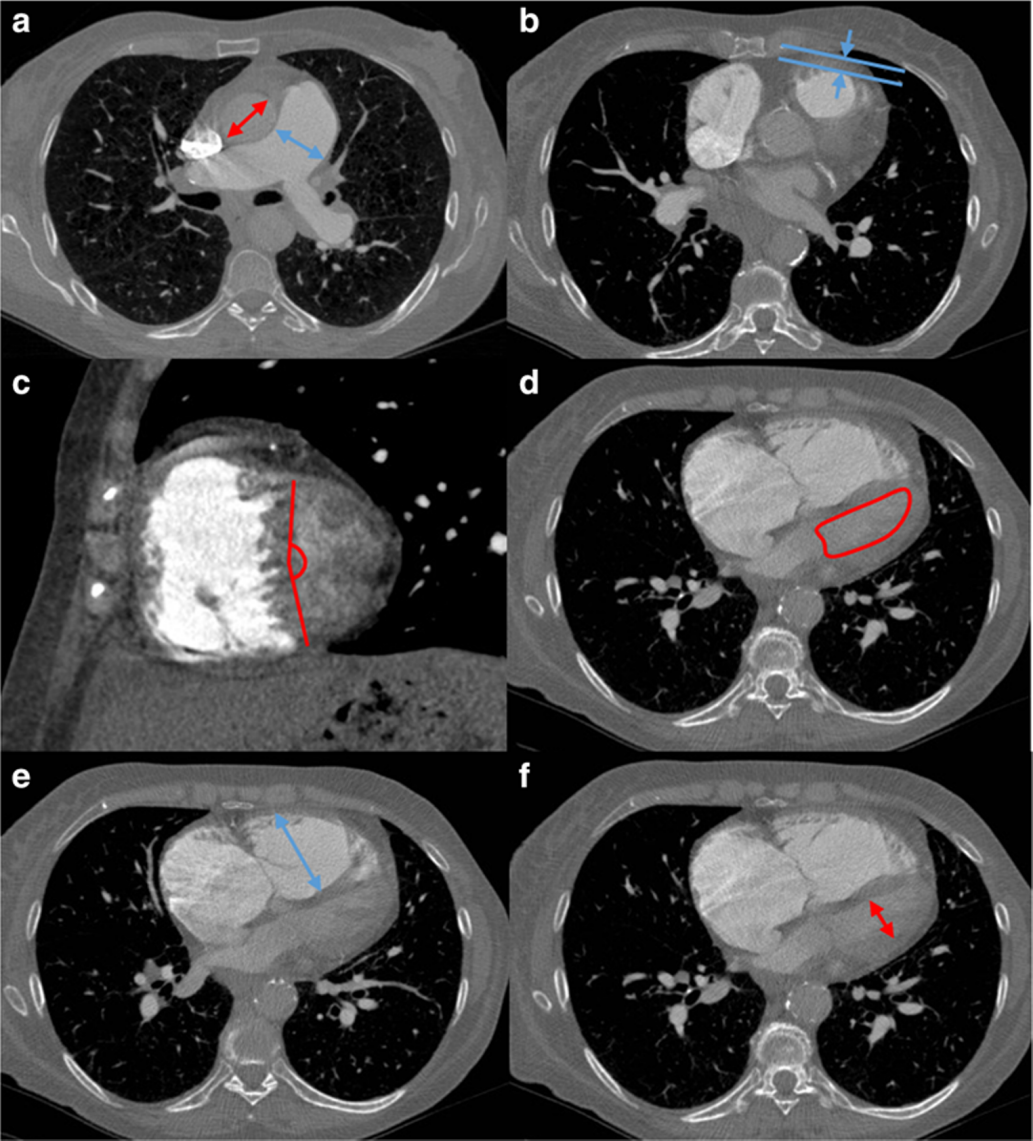
Multi-figure CT images illustrating the CT measurements in a patient with PH with severe elevation of pulmonary artery pressure. Illustrations show the measurement of pulmonary artery and aortic diameter (**a**), right ventricular outflow tract thickness (**b**), interventricular septal angle (**c**) (reconstructed short-axis images) and left ventricular area (**d**). Images **e** and **f** illustrate the measurements required to calculate the RV/LV diameter ratio maximal RV diameter (**e**) and maximal LV diameter (**f**)

Main pulmonary artery (PA) diameter was measured perpendicular to the vessel axis at the widest point. At the same level as the main PA measurement, the diameter of the ascending and descending aorta was recorded and the pulmonary artery to aortic ratio was calculated (Fig. 1a). Right and left main pulmonary artery diameters were measured at the widest point. The IVC diameter was measured just below the entry level to the right atrium. The extent of hepatic reflux was measured using an adapted 4 grade score of regurgitation.

#### Cardiac measurements

Maximal biventricular areas and diameters (mid-ventricular) and atrial areas were manually traced on axial images. Slices were chosen to ensure the greatest area or diameter was measured. Ventricle and atrial diameters were measured on the same slices as area measurements. Ventricular muscle area and circumference walls were measured for both right and left ventricles. The muscular thickness of the RV outflow tract was measured anteriorly ~ 1 cm below the pulmonary valve (Fig. 1b). Maximal LV area is shown in Fig. 1d and maximal RV and LV diameters are shown in Fig. 1e and f respectively.

#### Reconstructed short-axis images

Images were reconstructed using multi-planar reformat software (IMPAX, volume viewer, Agfa HealthCare) to generate a mid-chamber short-axis image and 4-chamber image. On the short-axis image, the interventricular septal angle was measured, defined as the angle from the RV insertion points to the mid-interventricular septum (Fig. 1c). On the reconstructed 4-chamber image, biventricular areas and diameters (mid-ventricular) and atrial areas were manually traced.

MR image acquisition and analysis methods and details on right heart catheterisation are found in the Supplementary Methods.

### Statistics

Normal distribution was assessed through visual and statistical analysis, using histograms and quantile-quantile (Q-Q) plots as a way of determining normality of all variables. Where appropriate, continuous data was presented with mean and standard deviation. Group comparison of continuous data was made using independent *t* test and categorical group comparisons was made using chi-square and Fisher’s exact test. Correlations between CT measurement and mPAP, PVR and cardiac MRI data were made using Pearson’s correlation test. Derivation and validation cohorts were identified using random number generation. CT variables significant at independent *t* test or chi-square at univariate analysis (*p* < 0.05) in the derivation cohort were entered into a binary logistic regression model using forward stepwise selection. CT diagnostic model A was the resultant model. Sensitive, compromise and specific diagnostic thresholds were identified in the derivation cohort by visual inspection of ROC curves. In the validation cohort, the derived thresholds were tested using the 2 × 2 contingency table to determine sensitivity, specificity and positive and negative predictive values. A second model, CT diagnostic model B, was developed without septal angle; septal angle is the only measurement that requires image reconstruction, and some observers will not have access to a reconstruction tool when reporting or in situations where there is limited time; therefore, we elected to develop a model that could be used on the axial images alone.

The prognostic value of CT sensitive, compromise and specific diagnostic thresholds and right heart catheterisation thresholds of ≥ 25 mmHg and > 20 mmHg was assessed using Kaplan-Meier and Cox proportional hazards regression analysis. The intra-class correlation co-efficient (ICC) was used to test the reproducibility of CT metrics. IBM SPSS Statistics 22 was used to perform the statistical analysis. A *p* value of < 0.05 was considered statistically significant.

## Results

**Fig. 2:**
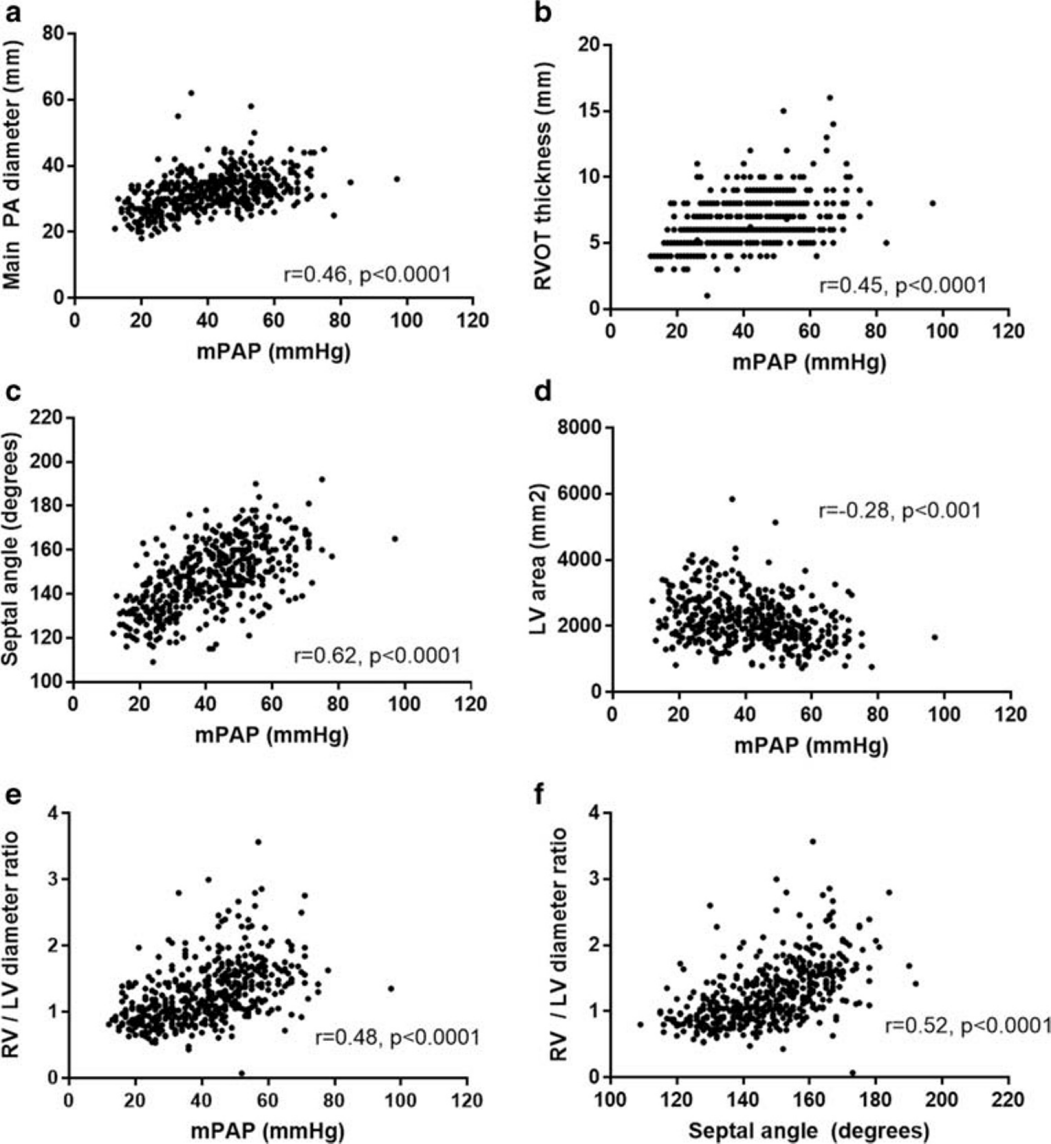
Correlations of mean pulmonary arterial pressure with main pulmonary artery diameter (**a**), right ventricular outflow tract thickness (RVOT) (**b**), interventricular septal angle (**c**), left ventricular (LV) area (**d**) and RV/LV diameter ratio (**e**). **f** Correlation between septal angle and RV/LV diameter ratio

Between April 2012 and March 2016, 840 consecutive treatment-naïve patients with suspected PH were identified who underwent MRI and RHC, of whom 491 patients underwent CT pulmonary angiography within 90 days. Patients underwent CT imaging at 68 different institutions and 78% of CT pulmonary angiograms were performed at the Sheffield Pulmonary Vascular Disease Unit. Patient demographics, RHC and CT metrics for patients with PH (*n* = 420), with mPAP < 25 mmHg (*n* = 71) and with mPAP ≤ 20 mmHg are shown in Table 1. Patients with PH were older (*p* < 0.001) and more likely to be female (*p* < 0.013) and have a higher WHO functional class (*p* < 0.001) and lower walking distance (*p* < 0.001), than patients without PH. Correlation of CT metrics with mPAP and PVR is presented in Supplementary Table 1 and key correlations are in Fig. 2. Table 2 presents the sensitivity, specificity and positive and negative predictive value of pulmonary artery diameter, right ventricular outflow tract thickness, interventricular septal angle and RV/LV diameter ratio.

**Table 1:** Demographics of patients with and without PH for the full cohort

Covariates	No PH, mPAP ≤ 20 mmHg, *n* = 36	No PH, mPAP < 25 mmHg, *n* = 71	PH, mPAP ≥ 25 mmHg, *n* = 420	*p* value < 25 vs ≥ 25 mmHg
Demographics				
Age (years)	61 (14)	60 (15)	65 (13)	< 0.001
Sex % female		69%	58%	0.013
BSA (m^2^)	1.8 (0.2)	1.86 (0.27)	1.83 (0.23)	0.377
WHO FC % (1/2/3/4)	0/18/17/1	0/49/50/1	0/7/83/10	< 0.001
ISWT				
Distance (m)	346 (211)	315 (203)	206 (187)	< 0.001
RHC				
mRAP (mmHg)	5 (3)	6 (3)	11 (6)	< 0.001
mPAP (mmHg)	17 (2)	20 (3)	45 (13)	< 0.001
PAWP (mmHg)	9 (5)	11 (5)	13 (5)	< 0.001
Cardiac output (L/min)	5.5 (1.4)	5.7 (1.5)	4.8 (1.5)	< 0.001
Cardiac index (L/min/m^2^)	3.0 (0.8)	3.1 (0.8)	2.6 (0.8)	< 0.001
PVR (dyns)	120 (1250)	138 (102)	603 (385)	< 0.001
Computed tomography				
Right heart metrics				
RA area	2085 (659)	2263 (1152)	3108 (1144)	< 0.001
RV diameter (mm)	36 (7.7)	38 (8.1)	45 (9.1)	< 0.001
RV muscle wall area (mm^2^)	247 (114)	272 (117)	416 (184)	< 0.001
RV chamber area (mm^2^)	2084 (658)	2272 (766)	3005 (918)	0.011
RV outflow tract (mm)	4.8 (1.2)	4.9 (1.4)	7.0 (1.9)	0.004
Septal angle (degrees)	129 (7.2)	132 (9.5)	151 (14.2)	< 0.001
Left heart metrics				
LA area (mm^2^)	1967 (479)	2095 (632)	2165 (768)	0.47
LV chamber area (mm^2^)	2256 (656)	2405 (707)	2123 (704)	0.002
LV muscle area (mm^2^)	1549 (357)	1618 (396)	1586 (450)	0.584
Ratios				
RA area/LA area ratio	1.0 (0.3)	1.1 (0.39)	1.6 (1.46)	0.003
RV diameter/LV diameter	1.0 (0.2)	0.95 (0.25)	1.33 (0.46)	< 0.001
RV muscle area/LV muscle area	0.16 (0.08)	0.17 (0.77)	0.28 (0.14)	< 0.001
RV chamber area/LV chamber area	1.0 (0.3)	0.99 (0.38)	1.56 (0.75)	< 0.001
Vessel measurements				
Main PA diameter (mm)	26 (4)	26 (4.0)	33 (5.3)	0.07
Main PA/ascending aorta ratio (ratio)	0.84 (0.13)	0.87 (0.17)	1.05 (0.19)	< 0.001
Main PA/descending aorta ratio (ratio)	1.17 (0.20)	1.2 (0.22)	1.43 (0.30)	< 0.001
Left PA diameter (mm)	21.1 (4.1)	22 (3.6)	25 (3.7)	< 0.001
Right PA diameter (mm)	21.9 (4.3)	22 (4.4)	26 (4.4)	< 0.001
Inferior vena cava area (mm^2^)	525 (144)	579 (209)	639 (207)	0.023
Superior vena cava area (mm^2^)	305 (80)	320 (104)	382 (134)	< 0.001
Hepatic reflux of contrast (score 0 to 3)	0 (18), 1 (13), 2 (3), 3 (1)	0 (35), 1 (24), 2 (7) and 3 (4)	0 (128), 1 (117), 2 (98) and 3 (73)	< 0.001

*BSA*, body surface area; *WHO FC*, World Health Organisation functional class; *mRAP*, mean right atrial pressure; *mPAP*, mean pulmonary arterial pressure; *PAWP*, pulmonary arterial wedge pressure; *PVR*, pulmonary vascular resistance; *RA*, right atrium; *RV*, right ventricle; *LA*, left atrium; *LV*, left ventricle; *PA*, pulmonary artery

**Table 2:** Diagnostic accuracy of predictive thresholds for PA diameter, RVOT thickness, septal angle and RV diameter/LV diameter ratio in the validation cohort

Thresholds	Sensitivity	Specificity	Positive predictive value	Negative predictive value	*p* value
PA diameter					
Sensitive ≥ 28 mm	90	71	95	50	< 0.001
Compromise ≥ 30 mm	75	81	96	32	< 0.001
Specific ≥ 32 mm	58	90	98	24	< 0.001
RVOT thickness					
Sensitive ≥ 5 mm	95	42	92	57	< 0.001
Compromise ≥ 6 mm	80	77	96	36	< 0.001
Specific ≥ 7 mm	55	97	99	24	< 0.001
Septal angle					
Sensitive ≥ 130	93	35	91	42	< 0.001
Compromise ≥ 140	78	97	99	39	< 0.001
Specific ≥ 150	51	97	99	22	< 0.001
RV diameter/LV diameter					
Sensitive ≥ 0.8	89	19	88	20	0.2375
Compromise ≥ 1	72	58	92	24	0.0015
Specific ≥ 1.2	50	90	97	21	< 0.001

*PA*, pulmonary artery; *RVOT*, right ventricle outflow tract; *RV*, right ventricle; *LV*, left ventricle

### Derivation cohort

Random patient selection identified a derivation cohort of 247 and a validation cohort of 244 patients. There were no significant differences in age, proportion of patients with PH, WHO functional class or right heart catheterisation metrics between the two cohorts (*p* > 0.05). However, there was a higher proportion of females in the derivation cohort as compared with the validation cohort (Supplementary Table 2).

#### CT diagnostic model A

**Fig. 3:**
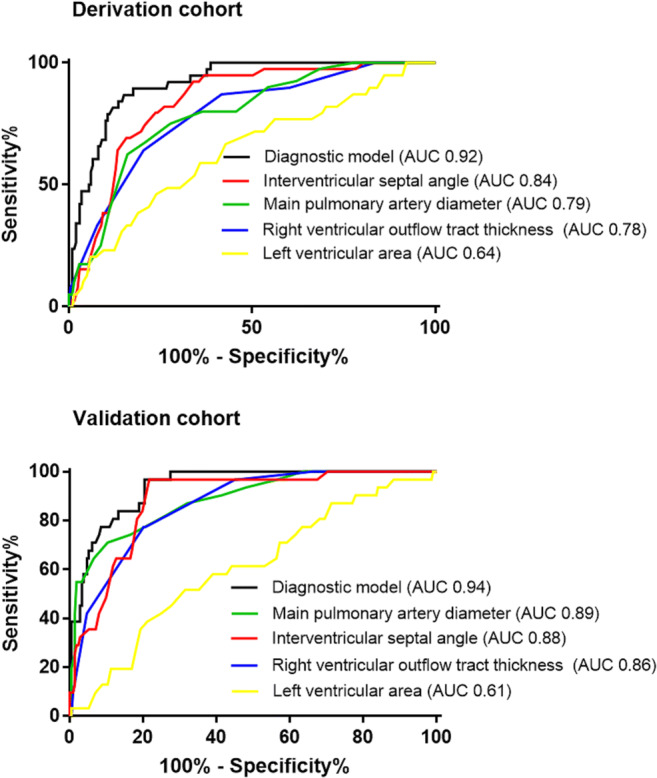
Receiver operating characteristic analysis in a derivation cohort and validation cohort for diagnostic model A for prediction of mPAP ≥ 25 mmHg

In the derivation cohort, a regression model was identified. The model incorporated main pulmonary artery diameter, right ventricle outflow tract thickness, left ventricular area and interventricular septal angle as follows: model A score = − 14.299 + (0.192 × main pulmonary artery diameter, mm) + (0.518 × right ventricle outflow tract thickness, mm) − (0.001 × left ventricular area, mm^2^) + (0.068 × interventricular septal angle, degrees). The area under the curve (AUC) in the derivation cohort was 0.92 (see Fig. 3). The AUC in the derivation cohort with adjustment for body surface area was 0.86. The following thresholds were identified in the derivation cohort: high sensitivity (model A score 0), high specificity (model A score 2.5) and a compromise threshold (model A score 1.25). The diagnostic model performed better than individual CT metrics. Of the individual CT metrics, the AUC for pulmonary artery diameter was 0.79, right ventricular outflow tract thickness 0.79, left ventricular area 0.64 and interventricular septal angle 0.84.

For the prediction of mPAP > 20 mmHg, a model of − 13.843 + (0.94 × right ventricle outflow tract thickness, mm) + (0.094 × interventricular septal angle, degrees) was identified. The diagnostic accuracy of this model was AUC 0.88 for detecting mPAP > 20 mmHg; this was of lower accuracy in comparison with model A that had a diagnostic accuracy of 0.90 for detecting mPAP > 20 mmHg.

#### CT diagnostic model B

In the derivation cohort, a second model was developed; the model incorporated main pulmonary artery diameter, right ventricle outflow tract thickness and RV/LV diameter ratio, as follows: model B score = − 9.181 + (0.174 × main pulmonary artery diameter, mm) + (0.480 × right ventricle outflow tract thickness, mm) + (2.539 × RV/LV diameter, ratio). This model had an AUC of 0.89 in the derivation cohort. The following thresholds were identified in the derivation cohort: high sensitivity (model B score 0.5), compromise threshold (model B score 1.0) and a high specificity threshold (model B score 1.5) (see Table 4). CT diagnostic prediction model to detect mPAP > 20 mmHg was also derived: − 4.553 + (0.661 × right ventricle outflow tract thickness, mm) + (3.027 × RV/LV diameter, ratio). This model had lower accuracy for prediction of mPAP > 20 mmHg at ROC analysis: AUC 0.86 compared with 0.89 for model B.

### Validation cohort

#### Identification of patients with mPAP greater than or equal to 25 mmHg

##### CT diagnostic model A

In the validation cohort, the CT diagnostic model A showed high diagnostic accuracy for the detection of PH (AUC at 0.94; Fig. 3). The CT diagnostic model A adjusted for BSA did not improve the diagnostic performance of the model (AUC 0.92). Sensitivity, specificity and positive and negative predictive values are presented for high sensitivity, specificity and compromise thresholds in Table 3.

**Table 3:** Regression CT diagnostic models A and B thresholds and their accuracy for predicting the presence of PH in the validation cohort

Thresholds	Sensitivity	Specificity	Positive predictive value	Negative predictive value	*p* value
Diagnostic model A					
Sensitive ≥ 0 units	96	58	94	69	< 0.001
Compromise ≥ 1.25 units	82	84	97	41	< 0.001
Specific ≥ 2.5 units	71	100	100	33	< 0.001
Diagnostic model B					
Sensitive ≥ 0.5 units	92	71	96	55	< 0.001
Compromise ≥ 1 units	84	81	97	42	< 0.001
Specific ≥ 1.5 units	75	90	98	35	< 0.001

##### CT diagnostic model B

Model B was derived excluding the single parameter that required reconstruction (interventricular septal angle). In the validation cohort, diagnostic CT model B had an accuracy of 0.92. Table 3 details the sensitivity, specificity and positive and negative predictive values for high sensitivity, specificity and compromise thresholds.

#### Identification of patients with mPAP greater than 20 mmHg

##### CT diagnostic model A

In the validation cohort, the CT diagnostic model A showed high diagnostic accuracy for the detection of PH (AUC at 0.91). The CT diagnostic model A adjusted for BSA marginally improved the diagnostic performance of the model (AUC 0.93).

##### CT diagnostic model B

Model B was derived excluding the single parameter that required reconstruction (interventricular septal angle). In the validation cohort, diagnostic CT model B was marginally weaker than model A with an accuracy of 0.87.

Table 4 details the sensitivity, specificity and positive and negative predictive values for high sensitivity, specificity and compromise thresholds for identification of patients with mPAP greater than or equal to 20 mmHg.

**Table 4:** Regression CT diagnostic models A and B thresholds and their accuracy for predicting the presence of patients with mPAP greater than 20 mmHg in the validation cohort

Thresholds	Sensitivity	Specificity	Positive predictive value	Negative predictive value	*p* value
Diagnostic model A					
Sensitive ≥ 0 units	93	67	98	38	< 0.001
Compromise ≥ 1 units	82	80	98	22	< 0.001
Specific ≥ 2.25 units	67	100	100	17	< 0.001
Diagnostic model B					
Sensitive ≥ 0.5 units	87	67	98	25	< 0.001
Compromise ≥ 0.8 units	82	90	98	23	< 0.001
Specific ≥ 1.4 units	72	80	98	16	< 0.001

#### Prognostic significance of CT and right heart catheterisation thresholds

##### CT diagnostic model A

In the validation cohort, 93 patients died; mean follow-up was 42 months. The CT diagnostic model A sensitive (0), compromise (1.25) and (2.5) specific thresholds for mPAP ≥ 25 mmHg were strongly predictive of mortality log rank 11.13 (*p* = 0.0009 and 9.70; *p* = 0.002 and 9.49; *p* = 0.002 respectively). The CT diagnostic model A sensitive (0), compromise (1.0) and (2.25) specific thresholds for mPAP > 20 mmHg were also strongly predictive of mortality log rank 11.13 (*p* = 0.0009 and 6.25; *p* = 0.010 and 10.57; *p* = 0.001).

##### CT diagnostic model B

The CT diagnostic model B sensitive (0.5), compromise (1.0) and (1.5) specific thresholds for mPAP ≥ 25 mmHg were as follows (mortality log rank 6.92): *p* = 0.009 and 3.25; *p* = 0.071 and 6.28; *p* = 0.012 respectively. The CT diagnostic model B sensitive (0.5), compromise (0.8) and (1.4) specific thresholds for mPAP > 20 mmHg were also strongly predictive of mortality log rank 6.92 (*p* = 0.009 and 6.56; *p* = 0.010 and 535; *p* = 0.021).

At Cox regression analysis, CT diagnostic models A and B were prognostic; *z* score hazard ratios were 1.56 and 1.42, both *p* < 0.0001.

##### Right heart catheter diagnostic thresholds

**Fig. 4:**
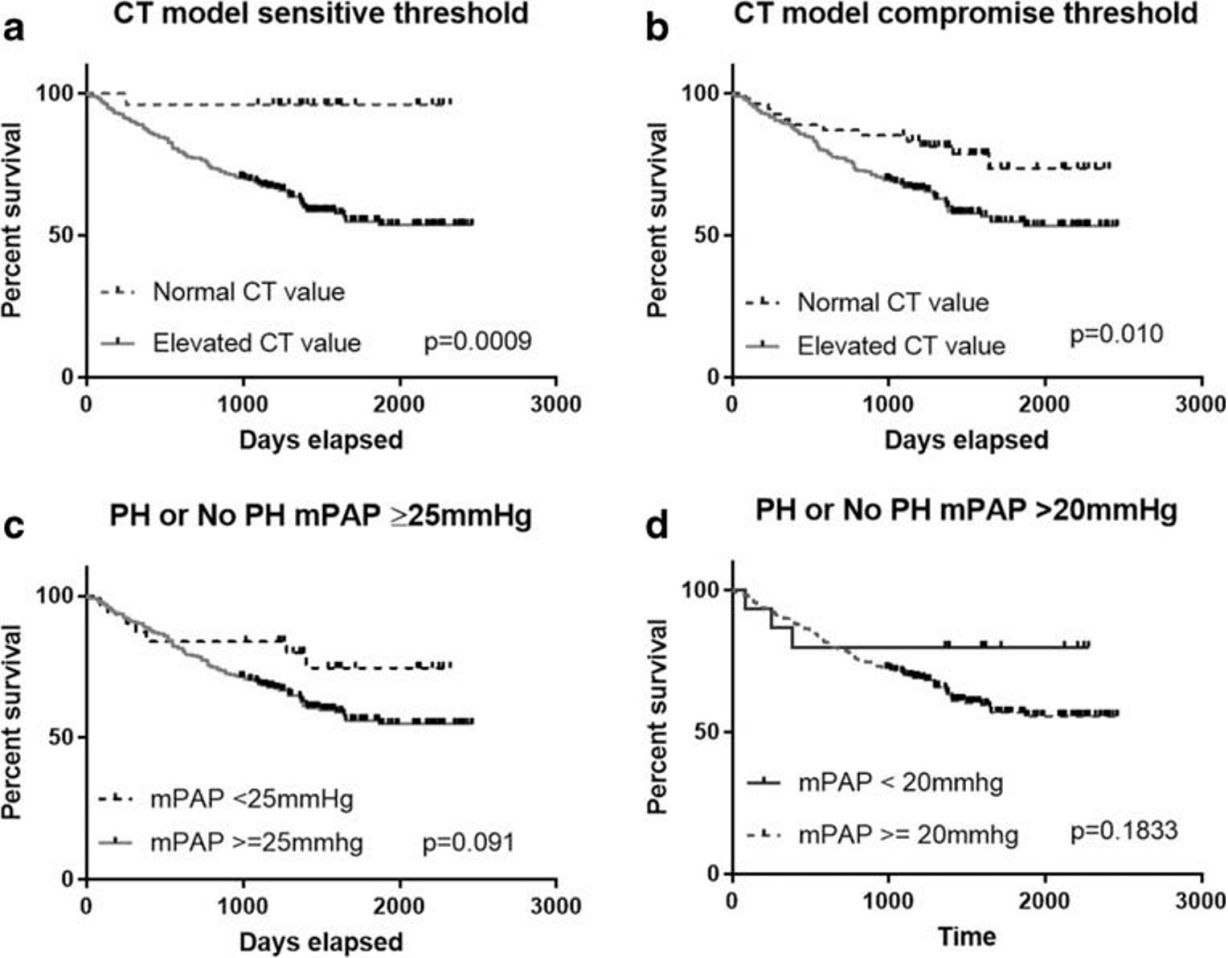
Prognostic significance of CT model A showing CT thresholds ((**a**) sensitive threshold and (**b**) compromise threshold) and mPAP thresholds ((**c**) mPAP ≥ 25 mmHg and (**d**) mPAP > 20 mmHg)

RHC diagnostic thresholds ≥ 25 mmHg and > 20 mmHg were not prognostic in this cohort (log rank 2.86, *p* = 0.09 and log rank 1.77, *p* = 0.18 respectively; see Fig. 4).

### Correlations and diagnostic value of individual CT metrics with pulmonary haemodynamics and MRI metrics in the full cohort

Correlations between CT vascular and cardiac measures are shown in Supplementary Table 1. Figure 2 shows the correlation of CT metrics and mean pulmonary artery pressure. A detailed description of the MRI findings is found in Supplementary Results.

### Reproducibility

High reproducibility of interventricular septal angle (ICC 0.921), pulmonary artery diameter (ICC 0.954) and left ventricular area (ICC 0.953) was demonstrated. In comparison, good reproducibility was recorded for the variables RV/LV diameter ratio (ICC 0.810) and right ventricular outflow tract thickness (ICC 0.76) (see Table 5).

**Table 5:** Reproducibility tests of the variables selected in model

Covariates	Intra-class correlation	Upper 95% CI	Lower 95% CI	*p* value
Septal angle	0.921	0.973	0.769	< 0.001
PA diameter	0.954	0.988	0.710	< 0.001
RV outflow tract	0.760	0.920	0.263	0.007
LV area	0.953	0.984	0.862	< 0.001
RV/LV diameter ratio	0.810	0.447	0.936	0.002

*PA*, pulmonary artery; *RV*, right ventricle; *LV*, left ventricle

## Discussion

In this study, we have shown that CT diagnostic models combining multiple metrics are superior to individual metrics in predicting the likelihood of PH. We have created models that utilise axial and reconstructed images and have also developed pragmatic scoring systems based on axial only images to improve the accessibility of CT to both radiologists and physicians. This approach could be used to increase or reduce the pre-test probability of PH. In addition, the CT diagnostic model had prognostic value with a negative score particularly at the sensitive threshold, indicating excellent survival.

Regression analysis identified pulmonary artery diameter, right ventricular outflow tract thickness, left ventricular area and interventricular septal angle as having additive value for the diagnosis of PH (CT diagnostic model A). Using a threshold of ≥ 0 units had a sensitivity of 96% and specificity of 58%, whereas a threshold of ≥ 2.5 units had a sensitivity of 71% and specificity of 100% in the validation cohort. An alternative model (CT diagnostic model B), utilising measurements from axial images alone, pulmonary artery diameter, right ventricular outflow tract thickness and the RV/LV diameter ratio, although marginally weaker also had good diagnostic accuracy. This model may provide a practical alternative if reconstruction tools are not available when reviewing CT images. In this model, a score of ≥ 0.5 units had a sensitivity of 92% and specificity of 71%, whereas a score of ≥ 1.5 units had a sensitivity of 75% and specificity of 90%.

The most commonly measured vessel on CT pulmonary angiograms in suspected PH is the pulmonary artery. The Framingham study established a reference range for normal and established 27 mm and 29 mm as representing the upper limits of normal for female and male patients, respectively. Previous studies using pulmonary artery size to diagnose PH have shown that a pulmonary artery diameter greater than 29 mm had a sensitivity of 75% and specificity of 89% for the presence of PH [29]. The utility of measuring the main pulmonary artery diameter and the pulmonary artery to aortic ratio has also been studied in suspected PH. Ng et al found that a pulmonary artery to aortic ratio > 1 was 92% specific for a mPAP > 20 mmHg [30]; other reports indicate diagnostic value in suspected PH [31, 32]. However, the pulmonary artery may be enlarged in the absence of PH and increases in pulmonary artery size over time are a feature of PH and are not necessarily an indication of increasing pulmonary artery pressure; as such, the correlation with mPAP is weak [33]. Although some investigators have suggested that pulmonary artery diameter may be unreliable in patients with underlying interstitial lung disease [34], we have recently shown that PA size has equivalent diagnostic utility in all patients with suspected PH and interstitial lung disease [35]. The present study confirms pulmonary artery diameter as an independent predictor of the presence of PH. We identified three thresholds: ≥ 28 mm as sensitive, ≥ 30 mm as a compromise and ≥ 32 mm as a specific threshold. These three thresholds can be used depending on the clinical scenario. Of CT measures used, the pulmonary artery diameter was the most reproducible. PA diameter, however, did not prove to be a significant independent predictor of patients with PH defined by mPAP ≥ 20 mmHg. We suspect this is due to underpowering based on the small number of patients with mPAP < 20 mmHg; further work in populations with larger number of patients with mild disease is required to better develop a predictive model for identification of patients with mPAP ≥ 20 mmHg.

Interventricular septal angle also showed strong diagnostic value and, when increased, had high specificity for the presence of PH. In the present study, CT images were reconstructed into the short-axis plane and a moderate correlation was identified with mPAP (*r* = 0.62), though weaker than that identified previously using MRI-derived systolic septal angle (*r* = 0.82) [36]. However, in this population, the diagnostic accuracy of CT septal angle was similar to that of MRI-derived systolic septal angle [11], which was an unexpected finding given the non-gated nature of CT. This may reflect the impact of RV enlargement and pressure overload both in diastole and systole which is seen frequently in the setting of significant pre-capillary disease. However, gating may be more important when minor elevations of pulmonary artery pressure are being investigated. Min et al also studied CT septal angle using ungated CT pulmonary angiography demonstrating a close correlation with pulmonary vascular resistance with high accuracy for detecting elevated PVR [21]. Septal angle may also have a role in the identification of patients with combined pre- and post-capillary PH, which has been demonstrated using MRI [37]. The ratio of the right ventricular to left ventricular diameter has prognostic value in PAH [17] and we have also demonstrated diagnostic value in this study. However, using reconstructed short-axis images to calculate septal angle, rather than using this ratio, improved diagnostic certainty. The ratio of the right ventricular to left ventricular diameter, however, still provides additional diagnostic value when added to pulmonary artery size and right ventricular outflow tract diameter and should be combined with these measures, when images cannot be reconstructed, to improve the diagnostic performance of CT pulmonary angiography.

The musculature of the right ventricular outflow tract is compacted and subjectively easier to measure than the trabeculated right ventricular free wall. Hence, it is not unexpected that the outflow tract thickness had higher diagnostic accuracy. Nonetheless, of all the metrics used in the diagnostic models, the right ventricular outflow tract thickness was the least reproducible. Right ventricular mass measured by MRI has also been shown previously to increase with pulmonary arterial pressure [9, 10]. This study has also examined the correlation of CT metrics with pulmonary haemodynamics. Septal angle had the highest correlation with mean pulmonary artery pressure of any of the CT metrics (*r* = 0.62) and was superior to RV/LV diameter ratio, demonstrating the value of reconstructing images.

### Limitations

The CT scans were not cardiac-gated, but despite this, CT measurements still had diagnostic value. Cardiac-gated CT allows for imaging of specific stages during a cardiac cycle and reduces cardiac motion artefacts seen in a CTPA and would help capture maximal deviation of the interventricular septum and volumetric metrics in future studies. Positive and negative predictive values will differ depending on the diagnostic setting; here, we show data from a tertiary referral centre population of patients with PH with a high pre-test probability of PH. This data may be particularly helpful when triaging patients with suspected severe PH for consideration of targeted pulmonary vascular therapies, although the diagnostic performance of CT in a community diagnostic setting has not been assessed.

## Conclusions

This study has developed and validated predictive thresholds using a combination of CT metrics in separate derivation and validation cohorts. Sensitive and specific thresholds have been identified that may be of value in both screening and for more definitive diagnosis depending on the clinical scenario. The diagnostic CT thresholds are also of prognostic value; patients found not to have PH on CT have an excellent outcome. Given the widespread use of CT to investigate unexplained breathlessness, a more systematic approach to CT evaluation may improve PH diagnostic rates.

## Electronic supplementary material

ESM 1 (DOCX 25 kb).

## Abbreviations

ASPIRE: Assessing the spectrum of PH identified at a referral centre
CMR: Cardiac magnetic resonance
CTEPH: Chronic thromboembolic PH
CTPA: Computerised tomography pulmonary angiogram
ICC: Intra-class correlation co-efficient
MRI: Magnetic resonance imaging
PA: Pulmonary artery
PAH: Pulmonary artery hypertension
PH: Pulmonary hypertension
Q-Q: Quantile-quantile
RHC: Right heart catheter
ROC: Receiver operating characteristic
