# Supplementary material for: Diagnostic accuracy of CT pulmonary angiography in suspected pulmonary hypertension
Source: Eur Radiol. 2020 Apr 27;30(9):4918–29. doi: 10.1007/s00330-020-06846-1 (PMC7431437; doi:10.1007/s00330-020-06846-1)
Supplement: Supplementary file 1 — (DOCX 25 kb). [file 330_2020_6846_MOESM1_ESM.docx]

**Supplementary methods**

**MRI acquisition**

CMR imaging was performed on a 1.5T whole body scanner GE HDx (GE Healthcare), using an 8-channel cardiac coil with the patient supine. Long axis LV 2 chamber and 4 chamber CINE views were acquired. 4 chamber and short axis (SA) cine images were acquired using a retrospective cardiac gated multi-slice balanced steady state free precession (bSSFP) sequence. A stack of axial images in the SA plane with slice thickness of 8-10 mm were acquired, fully covering both ventricles from base to apex. The bSSFP sequence parameters were: TR 2.8 ms, TE 1.0ms, Flip angle of 50°, field of view 400 x 400mm, 256 x 256 matrix, 125 kHz bandwidth and slice thickness of 10mm.

**MR Image analysis**

Image analysis was undertaken on a GE Advantage Workstation 4.4 and GE Advantage Workstation ReportCard. Scans were defined as non-diagnostic when image quality significantly affected cardiac measurements or, volumetric analysis could not be accurately performed. Right ventricular endocardial and epicardial borders were carefully outlined. The interventricular septum was considered as part of the left ventricle. The myocardial volume for each slice was calculated by multiplying the area of the right ventricular wall by the slice thickness. The product of the sum total of the myocardial slice volumes for each ventricle and the density of myocardium (1.05 g/cm3) was used to estimate right ventricular mass. The left ventricular epicardial and endocardial borders on each end-diastolic short axis slice were also outlined, LV end diastolic mass was derived. Ventricular mass index (VMI) was defined as RV mass divided by LV mass. Maximal and minimal PA areas were measured, and relative area change (RAC) was defined by the following equation: (maximal area-minimum area)/minimum area. The inter-ventricular septum was assessed on the mid chamber short axis CINE cardiac images at the phase of maximal septal displacement. Inter-ventricular septal angle was measured by determining the angle between the midpoint of the inter-ventricular septum and the two hinge points. The MR observer was blinded to right heart catheter data.

**Right heart catheterisation**

RHC was performed using a balloon-tipped 7.5F thermodilution catheter (Becton-Dickinson). Right heart catheterization was usually performed via the internal jugular vein using a Swan-Ganz catheter. Features at RHC required to define PH were mean PA pressure (mPAP) greater than or equal to 25 mm Hg at rest. A second analysis of greater than 20mmHg was also performed. Pulmonary vascular resistance (PVR) was determined as follows: PVR = (mPAP-PAWP)/cardiac output. Cardiac output was measured by thermodilution technique.

**Supplementary results**

Main pulmonary artery diameter had strong diagnostic value on the full cohort (AUC=0.84, p<0.001), stronger than the ratio of PA diameter:aortic diameter (AUC=0.80, p<0001). Main pulmonary artery diameter on CT correlated strongly with MRI derived pulmonary artery area in systole (r= 0.81) and diastole (r=0.81). Similar diagnostic accuracy was identified for detection of pulmonary hypertension for MRI derived systolic pulmonary artery area (AUC 0.86) and diastolic pulmonary artery area (AUC 0.83) (all p<0.0001). Right ventricular end diastolic mass derived on MRI correlated moderately with right ventricular muscle area on CT (r=0.46, p<0.001) and modestly with RV outflow tract thickness (r=0.27, p<0.001). MRI derived ventricular mass index (right ventricular mass/ left ventricular mass) also correlated with CT RV/LV muscle area ratio (r=0.50, p<0.0001). MRI derived RV mass (AUC=0.81) and ventricular mass index (AUC=0.81) were only marginally more accurate than CT derived right ventricular mass indices, right ventricular outflow tract hypertrophy (AUC 0.80), RV/LV muscle area ratio (AUC 0.76) and RV muscle area (AUC 0.760) for the diagnosis of PH. CT derived interventricular septal angle correlated well with MRI systolic (r=0.712) and diastolic angles (r=0.59), respectively. RV:LV diameter ratio correlated moderately with MRI systolic (r=0.54) and diastolic angles (r=0.38), respectively. In addition, similar diagnostic accuracy was shown for CT derived septal angle (AUC=0.85) and MRI systolic (AUC=0.85) and diastolic angles (AUC=0.74), (all p<0.0001). Correlations between CT vascular and cardiac measures are shown in **Supplementary Table 1. Figure 2** shows the correlation of CT metrics and mean pulmonary artery pressure.

**Online supplement**

Supplementary table 1 - Pearson correlations of CT metrics versus mean pulmonary arterial pressure (mPAP) and pulmonary vascular resistance (PVR)

| Covariates | mPAP  R-value | P value | PVR  R-value | P value |
| --- | --- | --- | --- | --- |
| Right Heart Metrics |  |  |  |  |
| RV Diameter | 0.448 | <0.001 | 0.418 | <0.001 |
| RV Muscle Area | 0.481 | <0.001 | 0.402 | <0.001 |
| RV Area | 0.343 | <0.001 | 0.381 | <0.001 |
| RV Outflow Tract Thickness | 0.450 | <0.001 | 0.214 | <0.001 |
| RA area | 0.343 | <0.001 | 0.279 | <0.001 |
| Septal angle | 0.622 | <0.001 | 0.592 | <0.001 |
| Left Heart Metrics |  |  |  |  |
| LV Diameter | -0.313 | <0.001 | -0.417 | <0.001 |
| LV Area | -0.281 | <0.001 | -0.414 | <0.001 |
| LV Muscle Area | -0.085 | 0.062 | -0.239 | <0.001 |
| LA Area | -0.148 | 0.001 | -0.336 | <0.001 |
| Ratios |  |  |  |  |
| RV/LV Diameter | 0.482 | <0.001 | 0.568 | <0.001 |
| RV/LV Muscle Area ratio | 0.501 | <0.001 | 0.513 | <0.001 |
| RV/LV Chamber Area ratio | 0.482 | <0.001 | 0.585 | <0.001 |
| RA /LA Area Index ratio | 0.278 | <0.001 | 0.365 | <0.001 |
| Vessel Metrics |  |  |  |  |
| Main PA Diameter | 0.458 | <0.001 | 0.298 | <0.001 |
| Main PA/Ascending Aorta Ratio | 0.440 | <0.001 | 0.341 | <0.001 |
| Left PA Diameter | 0.342 | <0.001 | 0.201 | <0.001 |
| Right PA Diameter | 0.303 | <0.001 | 0.172 | <0.001 |
| IVC area | -0.275 | 0.285 | -0.275 | 0.481 |
| SVC area | -0.421 | <0.001 | -0.507 | <0.001 |
| Hepatic reflux of contrast | 0.316 | <0.001 | 0.345 | <0.001 |

RA, right atrium; RV, right ventricle; LA, left atrium; LV, left ventricle; PA, pulmonary artery; IVC inferior vena cava; SVC superior vena cava

**Supplementary table 2**

Derivation and validation cohorts

| Covariates | Derivation Cohort n=247 | Validation Cohort n=244 | P-value |
| --- | --- | --- | --- |
| Demographics |  |  |  |
| Age (years) | 65 (13) | 64 (13) | 0.382 |
| Sex M:F (percent female) | 11/136 (92%) | 88/156 (64) | 0.009 |
| NoPH/PH | 2.59 (0.535) | 3.04 (0.410) | 0.472 |
| WHO FC I / II / III / IV (n) | I (1), II (29), III (190), IV (21) | I (0), II (30), III (189), IV (21) | |
| Body Surface Area | 1.84 (0.24) | 1.84 (0.23) | 0.715 |
| Right Heart Catheterisation | |  |  |
| mRAP (mmHg) | 10 (6) | 10 (5) | 0.311 |
| mPAP (mmHg) | 41 (15) | 42 (15) | 0.476 |
| PAWP (mmHg) | 13 (5) | 13 (6) | 0.411 |
| Cardiac Index (L.min/m2) | 2.64 (0.78) | 2.7 (0.89) | 0.37 |
| PVR (dyns) | 510 (365) | 548 (421) | 0.832 |
| SvO_2_ (%) | 65.1 (8.4) | 64.6 (8.7) | 0.543 |

BSA, body surface area; WHO FC, world health organisation functional class; mRAP mean right atrial pressure; mPAP mean pulmonary arterial presure; PAWP, pulmonary arterial wedge pressure; PVR, pulmonary vascular resistance; SvO2, mixed venous oxygen saturation.
